# Enhanced neural sensitivity to brief changes of happy over angry facial expressions in preschoolers: A fast periodic visual stimulation study

**DOI:** 10.1111/psyp.14725

**Published:** 2024-11-18

**Authors:** Sandra Naumann, Mareike Bayer, Isabel Dziobek

**Affiliations:** ^1^ Berlin School of Mind and Brain Humboldt‐Universität Zu Berlin Berlin Germany; ^2^ Department of Psychology, Institute of Life Sciences Humboldt‐Universität Zu Berlin Berlin Germany

**Keywords:** development, ERP, facial expression processing, FPVS, preschool children

## Abstract

Across childhood, emotion perception from facial expressions has traditionally been studied with event‐related potentials (ERP). Here, we explored the novel fast periodic visual stimulation (FPVS) electroencephalography (EEG) approach to provide information about how brief changes in facial expressions are processed implicitly in young children's brains. Utilizing two FPVS tasks for the first time in preschoolers, we examined brain responses to (1) the discrimination of brief changes in facial expressions at maximum intensity and (2) thresholds for discrimination of gradual increasing facial expression intensities. Within a stream of neutral faces at 6 Hz, happy and angry faces were embedded with a frequency of 1.2 Hz. Additionally, children performed an emotion recognition task (ERT). Data were collected in the context of a training study for socio‐emotional competencies with typically developing children (*N* = 74; 5.1[0.9] years; 34 females). FPVS data were collected post‐training, where training was included as a controlling factor. Across FPVS tasks, we detected robust expression change responses, particularly with larger responses for happy versus angry faces in the maximum intensity task. ERT results paralleled neural findings with faster reaction times and higher accuracy rates for happy versus angry faces. For gradual increases in emotional intensity, we found linear increases in responses across emotions. The majority of the sample showed a significant expression change at 60% intensity. With its implicit nature, short duration, and robustness of individual responses, our results highlight the potential of FPVS in comparison to classical ERP methods to study neural mechanisms of emotion perception in preschool samples.

## Abstract 2

Employing the fast periodic visual stimulation (FPVS) EEG approach, we detected a reliable index of emotional expression change with enhanced processing for happy compared to angry faces in young children (4–6 years). Our findings highlight the potential of this method to detect the processing of expression change implicitly in a short amount of time.

## 1 INTRODUCTION

The development of more complex socio‐emotional competencies, including empathy (Trentacosta & Fine, 2010; Zajdel et al., 2013) and prosocial behavior (Brazzelli et al., 2022), is predicated on the ability to identify and comprehend the emotions of others (Beauchamp & Anderson, 2010). Preschool years (ages 3–6) are fundamental to the development of emotion recognition as they offer an abundance of social learning opportunities (Denham, 2018; Denham et al., 2009). The ability to distinguish between emotions is still developing within this particular age group (Camras & Halberstadt, 2017; Weigelt et al., 2014): Previous research on explicit emotion detection tasks demonstrated that 5‐ to 6‐year‐old preschoolers were able to accurately identify happy facial expressions at a level similar to adults (Durand et al., 2007). However, their ability to perceive negative facial expressions was less accurate (Gao & Maurer, 2010).

Electroencephalography (EEG) has been previously used to study how preschoolers’ brains develop the ability to recognize emotions, especially from facial expressions (e.g., Curtis & Cicchetti, 2011; Naumann et al., 2022). Because of its easy, fast, and non‐intrusive applicability, it is especially suitable for populations with limited attention and a strong demand for physical activity (e.g., infants or young children). Early and late facial expression processing differences in preschoolers can be mapped with event‐related potentials (ERPs; Curtis & Cicchetti, 2011; D'Hondt et al., 2017; Naumann et al., 2022; Vlamings et al., 2010): In this age range, brain responses most commonly recorded in response to face, and expressive face stimuli were the ERP components P1 and N170 (Bhavnani et al., 2021). These early, sensory components are associated with the initial and automatic detection of facial features and structural face processing (peaks at 100 and 170 ms, respectively, Ding et al., 2017; Hinojosa et al., 2015). Higher‐order, later ERPs, such as the P3 component, are linked to in‐depth face processing (typically observed after 300 ms; Luo et al., 2010). Although results are heterogeneous, most studies that utilized implicit emotion recognition measures, such as passive face viewing paradigms, indicated that 3‐ to 6‐year‐old preschool children showed increased amplitudes of early and late components when exposed to positive and negative facial expressions, as compared to neutral expressions (Curtis & Cicchetti, 2011; Naumann et al., 2022; Vlamings et al., 2010). These findings, akin to studies with adults that use both explicit and implicit tasks for emotion recognition (Schindler & Bublatzky, 2020), indicate that the fundamental brain processes involved in facial expression processing are already established in preschoolers.

In aforementioned studies, static pictures are commonly employed to compare the neural patterns associated with different facial expressions. However, in everyday situations, facial expressions can briefly and constantly change (e.g., from a more neutral expression to a happy smirk). Thus, for a more comprehensive understanding of facial expression perception, research investigating the ability to detect brief facial expression changes in preschoolers is needed. The Fast Periodic Visual Stimulation approach (FPVS, Rossion et al., 2015) was recently tested in preschool, school, and adult samples to study neural changes in the detection of facial identities and expressions (Dzhelyova et al., 2017; Leleu et al., 2018; Lochy et al., 2019, 2020; van der Donck et al., 2019; Vettori et al., 2019).

Within an FPVS paradigm, a deviant (expressive) face is presented in a stream of (neutral) faces. Neutral and expressive faces are shown at different, but fixed frequencies (Rossion et al., 2015; e.g., base rate: stream of neutral faces at 6 Hz, interleaved with a stream of expressive facces at an expression change rate of 1.2 Hz). The FPVS approach assumes that stimuli presented periodically induce synchronization in the brain and, consequently, a response occurring at the same frequency (Adrian & Matthews, 1934), which can be quantified using an EEG steady‐state visual evoked potential (SSVEP; see Norcia et al., 2015, for review). Thus, when the differences between a neutral and an expressive face are detected by the brain, its response can be quantified at the same frequencies (base response for neutral faces; expression change response for expressive faces). The implicit response to the stimulation sequences constitutes a major advantage over explicit measures (e.g., response times or accuracy rates), which are frequently skewed in developmental samples due to processing speed issues, general inhibition, or an inability to comprehend the task (Maguire et al., 2014; van der Donck et al., 2019). Robust responses in FPVS data are already evident after brief stimulation periods (e.g., Dzhelyova et al., 2017) and quantifiable at the individual level (Leleu et al., 2018), presenting another advantage for younger populations who have natural constraints in attentional resources, frequently resulting in substantial trial loss in long EEG setups reaching up to 50% (e.g., due to movement, Leppänen et al., 2007). This adversely affects the signal‐to‐noise ratio (SNR) and, consequently, the integrity of the data (Luck, 2005). Furthermore, time windows for mean or peak amplitude analyses in ERP research are often defined post hoc by the experimenter, which hampers comparability across studies (Luck & Gaspelin, 2017). Within the FPVS approach, frequencies are determined before the experiment, corresponding to the presentation frequencies. Additionally, results of an FPVS task can be quantified in the frequency and time domain, with the latter enabling the analysis of temporal trajectories in a manner similar to that of ERP quantification procedures (Dzhelyova et al., 2017; Leleu et al., 2018).

First studies implementing FPVS to examine brief expression changes in adults show promising results: Dzhelyova et al. (2017) presented either upright or inverted streams of neutral faces interleaved with happy, fearful, or disgusted facial expressions. Across two experiments, they detected a reliable expression change response for all emotions. Concerning differences between emotions, one observation detectable in both experiments was a larger expression change response in parietal regions for happy faces in comparison to those exhibiting a negative valence (e.g., fear or disgust; Dzhelyova et al., 2017). In addition, FPVS signals could be translated into a triphasic response in the time domain, which was found to be comparable to an ERP response after the presentation of facial expressions (e.g., with peaks at the P1, N170, and P3 time windows). A second FPVS study involving adults examined facial expressions with increasing intensity to determine the threshold required to elicit significant expression change responses (Leleu et al., 2018). All displayed emotions (anger, disgust, happiness, fear, and sadness) produced notable expression change responses. Disgusted faces showed the largest expression change response, whereas sad faces elicited the lowest responses. In addition, frequency power and thus brain synchronization increased with increasing expression intensity. In line with previous research, the authors also detected a triphasic response within the time domain (Leleu et al., 2018). Expanding the study of Dzhelyova et al. (2017), they discussed potential mechanisms for each component. The first component (C1) was linked to low‐level face encoding (similar to the P1); the second component (C2) to perceptual face encoding (similar to the N170); and the third component (C3) to facilitate more elaborate processing and categorization of emotional facial expressions (similar to the P3).

When brief expression changes of fearful faces were assessed in 8‐ to 12‐year‐old boys, a reliable expression change response was detected (van der Donck et al., 2019). More importantly, boys with autism showed diminished responses than boys without autism, underscoring the potential of FPVS for clinical applications. The application of the FPVS method to 5‐year‐old preschoolers has thus far been limited to the visual discrimination of faces from objects or individual faces, specifically, the processing of face identities (Lochy et al.,  2020). A robust and dependable deviant response was identified in these studies, with greater responses observed in the right hemisphere (Lochy et al., 2020).

**FIGURE 1:**
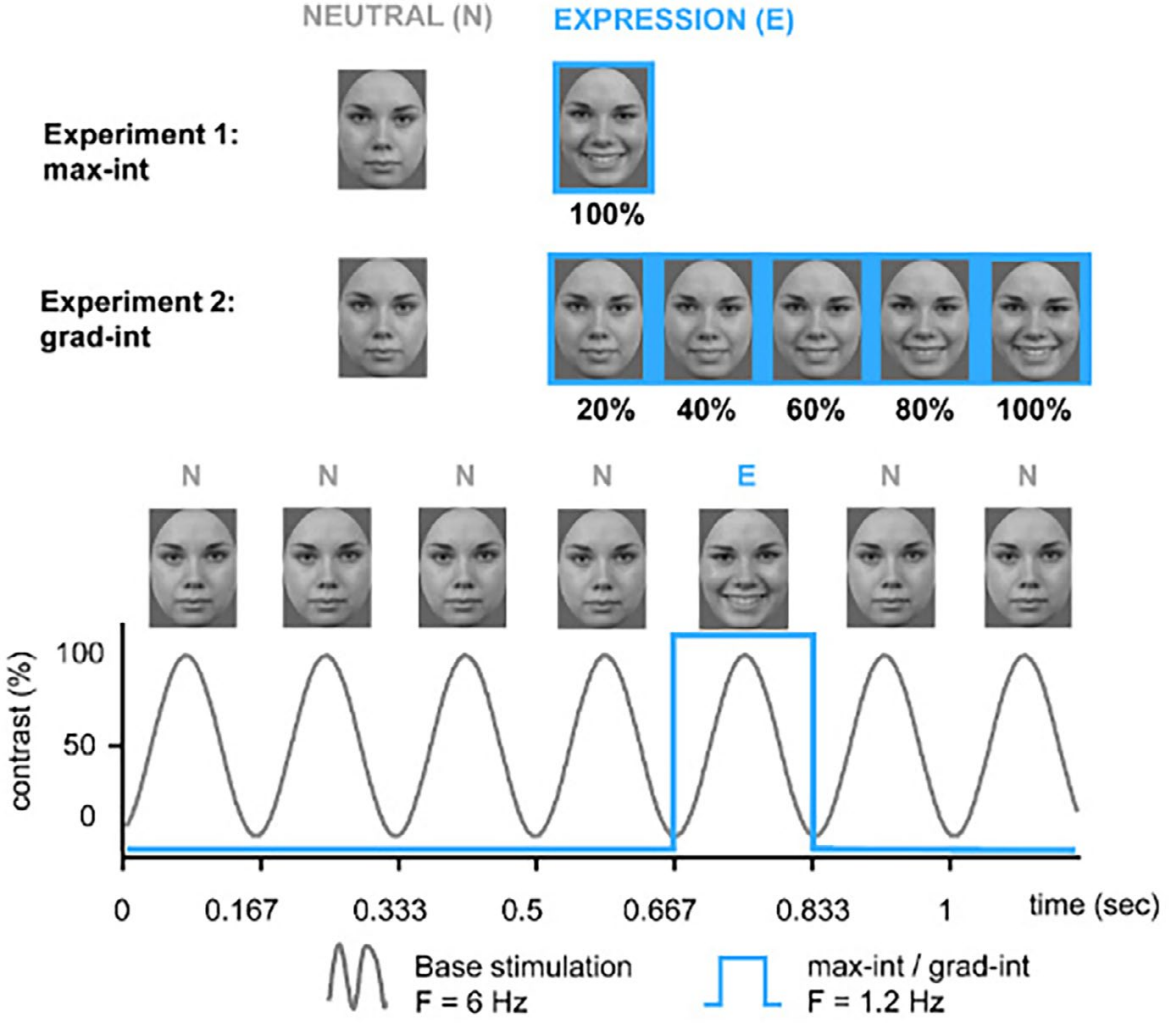
Fast periodic visual stimulation (FPVS) paradigms used in two separate tasks (max‐int and grad‐int).

In light of the previous literature, ERP research has established a critical foundation to examine the neural mechanisms underlying facial expression processing in preschoolers. Providing further insight into how the brain processes brief changes in facial expressions, the FPVS method could contribute to this body of research. First studies with adults and older children yield encouraging results; further research with younger samples is required. Hence, within our work, we employed the FPVS approach to examine preschoolers' brain responses to brief expression changes. Following previous FPVS procedures (Dzhelyova et al., 2017; Leleu et al., 2018; van der Donck et al., 2019), we employed two tasks (see Figure 1): The first task tested the discrimination of brief changes in facial expression with expressions at maximum intensity (hereafter “max‐int” task), and the second task examined the threshold for the discrimination of facial expressions with gradual increasing expression intensity (hereafter “grad‐int” task). In addition to the frequency‐domain analysis, we also examined time‐domain information (Dzhelyova et al., 2017; Leleu et al., 2018).

Firstly, we investigated whether preschoolers as previously shown for school‐aged children (8–12 years; van der Donck et al., 2019) and adults (Dzhelyova et al., 2017) can pick up brief changes in facial expression reliably, which would be indicated by significant responses to the expression change rate foremost within the max‐int task and the right hemisphere for younger populations (Lochy et al., 2020; Vettori et al., 2019). Secondly, we examined potential processing differences between emotions employing angry and happy faces. Previous studies on FPVS concerning emotion processing with adult samples and school‐aged children have not found a clear advantage of positive emotions over negative emotions (e.g., Dzhelyova et al., 2017; van der Donck et al., 2019). However, we hypothesize that in the case of preschoolers, happy faces would generate stronger signals than angry faces in the frequency and time domain within both FPVS tasks. This hypothesis is supported by prior ERP research (Naumann et al., 2023) and findings from behavioral studies which showed that happy faces are fastest and most accurately processed in young children (Durand et al., 2007; Gao & Maurer, 2010). Thirdly, we investigated potential threshold differences for increasing emotion intensities within the grad‐int task. In line with previous FPVS literature with adult samples (Leleu et al., 2018), we hypothesized that there is a linear increase in signal with increasing expression intensity in the expression change response as well as a linear decrease in the base response. Besides the two FPVS tasks, we also employed an explicit emotion recognition task (ERT) to measure how well preschoolers are able to distinguish neutral faces from expressive faces at different intensities. Relating to previous behavioral research with preschoolers (Durand et al., 2007; Gao & Maurer, 2010), we hypothesized a processing advantage for happy faces, detectable in higher accuracy rates as well as faster reaction times for happy compared to angry faces. In a more exploratory fashion, we examined individual expression change response trajectories for each participant (Leleu et al., 2018). We used a qualitative approach to detect potential individual variation as well as patterns across the sample.

The experimental design was part of a longitudinal study assessing the effectiveness of a social competence training program called *Zirkus Empathico* (findings from the training study were recently published: Naumann et al., 2023). In the training study, we hypothesized that the Zirkus Empathico group would show enhanced early and late ERP amplitudes (P1, N170, and P3), indicative of attentional resources dedicated to facial expression processing. In the present study, the effect of the training was considered as a control variable, the primary emphasis being on the neural processes involved in facial expression processing in preschool‐aged children, rather than the influence of training. The results can be found in the Supplementary Materials, and their implications are briefly mentioned in the discussion.

## 2 MATERIALS AND METHODS

The FPVS tasks were part of a pre‐registered training study (German register for clinical studies: DRKS‐ID: DRKS00015789); the study was approved by the local ethics committee. A comparative analysis was conducted between two digital training programs: Zirkus Empathico, which aimed to foster social–emotional competences in preschoolers, and Squirell & Bär, which focused on early foreign language acquisition via interaction with fundamental English words (see Supplement S1 for more information). The training study was pre‐registered with a sample size of 74 young children aged 4–6 years to provide 80% power at a two‐sided 5% α‐level (G*Power; Faul et al., 2007). In a post hoc analysis, we examined the achieved power to detect a change in expression change response within the max‐int and grad‐int task (R‐package mixedpower v0.1.0; Kumle et al., 2021). As a result, we detected an achieved power for a potential emotion effect of 86% for the max‐int task and 11% for the grad‐int task. Within the current analysis, we focused on the neural mechanisms of facial expression processing in preschoolers and not the modulation by training. Nevertheless, the factor training was integrated into all analyses to control for its potential effects and to compare with previous ERP findings of the same sample (results can be found in Supplement S3).

### 2.1 Participants

All participants were of Central European origin and between 4 and 6 years old (range: 4.0–6.9; *M* = 5.1, *SD* = 0.9). We also assessed children's non‐verbal IQ (colored progressive matrices, CPM; Raven, 2002) and verbal age (Peabody Picture Vocabulary Test, PPVT; Dunn & Dunn, 2007). To describe families' socio‐economic status (SES), we summarized family income, caregiver occupation, and education with the Winkler index (Winkler & Stolzenberg, 1998). Families' SES ranged from medium to high.

Regarding the FPVS data, we excluded participants due to (a) non‐compliance (*n* = 10, e.g., children became bored or agitated during the fast stimulation sequences and thus closed their eyes which led to non‐visibility of the base response), (b) heavy movement (*n* = 10, e.g., children tried to imitate the perceived expression change, which led to large muscle artifacts or non‐visibility of the base response), or (c) technical problems during data acquisition (*n* = 10, e.g., not all event triggers were recorded due to a software error). In addition, as max‐int and grad‐int conditions were recorded separately, not all children provided equally sufficient data for both conditions (e.g., heavy movements in one of the conditions), resulting in varying sample sizes for max‐int (*N* = 47) and grad‐int (*N* = 44). Compared to previous FPVS studies with younger and older children (e.g., attrition rates 4%–11%; Barry‐Anwar et al., 2018; Lochy et al., 2019, 2020; Vettori et al., 2020), our attrition rates were higher (max‐int: 37%; grad‐int: 41%). One explanation would be the duration of the paradigms which was doubled compared to previous studies with young samples (our study: 10 min and other studies: 4–5 min; e.g., Lochy et al.,  2020 or Vettori et al., 2020). In addition, we did not exclude trials with inadequate data quality for a participant (see, e.g., Barry‐Anwar et al., 2018, where approximately 50% of trials were removed per participant per task), but instead removed all of the participant's data from the analysis to ensure high data quality for remaining participants.

There were no differences between training and control groups in terms of their participant characteristics, which is why we provide concatenated information across groups separated for each FPVS experiment (see Table 1 for demographic and screening information; see Supplement S2: Tables S1 and S2 for demographic information separated by controls and the Zirkus Empathico group).

**TABLE 1:** Participant sociodemographic information for the max‐int and grad‐int task.

Characteristics	Max‐int (*N* = 47)	Grad‐int (*N* = 44)
Sex		
Female/Male	21/26	22/22
Age, *M* (*SD*)		
Years	5.3 (0.9)	5.3 (0.9)
SES (Winkler Index), *n* (%)		
Low	2 (4)	1 (2)
Medium	13 (28)	12 (27)
High	32 (68)	31 (71)
Verbal age, *M* (*SD*)		
PPVT percentiles	64.4 (27.3)	65.0 (27.2)
Non‐verbal IQ, *M* (*SD*)		
CPM score	15.1 (4.0)	15.3 (4.1)

Abbreviations: CPM, colored progressive matrices; PPVT, Peabody picture vocabulary test; SES, socio‐economic status (Winkler index; Winkler & Stolzenberg, 1998).

### 2.2 FPVS tasks

As shown in Figure 1, we used two FPVS tasks: The max‐int task tested the discrimination of brief changes of facial expression with expressions at maximum intensity and the grad‐int task examined the threshold for the discrimination of facial expressions with gradual increasing expression intensities. Both tasks were administered using Presentation® (Version 17.2, Neurobehavioral Systems, Inc., Berkeley, CA, www.neurobs.com).

#### 2.2.1 Stimuli

For max‐int and grad‐int, face stimuli consisted of happy, angry, and neutral facial expressions of two males and two females from the Radboud Faces Database (Langner et al., 2010). All stimuli were in full‐front view, gray scaled, adjusted to mean luminance, and trimmed to the same oval shape to exclude hair and non‐facial contours (height: 150 pixels, width: 110 pixels). All faces were presented on a gray background (RGB = 100, 100, and 100) using a 15″ monitor (display resolution: 1024 × 767) with a viewing distance of 70 cm (visual angle: 3.27°). For the grad‐int task, we generated facial morphs by parametrically merging neutral and expressive faces, increasing in five increments from 20% to 100% expression intensity (Morpheus Photo Morpher, Morpheus Software, 2014). To our knowledge, our study pioneered the FPVS paradigm examining brief expression change in preschoolers aged 4–6 years. Thus, we followed previous similar FPVS experimental setups for children and adults as closely as feasible (see Dzhelyova et al., 2017; Leleu et al., 2018; van der Donck et al., 2019). Within these studies, a limited number of face identities was presented, whereas other studies have used a greater variety of face stimuli (e.g., for review, see Peykarjou, 2022). However, we sought similarity to be able to compare our results to those studies. Secondly, young children naturally have limited attentional and cognitive capabilities. Thus, we (1) adhered to the FPVS protocol from previous studies to make it as comparable (for reproducibility purposes) to previous studies as possible and (2) kept it as short as possible to suit our study population's needs.

#### 2.2.2 Stimulation sequences

The max‐int and grad‐int tasks followed the same FPVS procedure (e.g., Leleu et al., 2018; van der Donck et al., 2019): Each sequence was preceded by a fixation cross displayed for 1000 ms. Subsequently, using a sine wave stimulation, a stream of neutral faces (N) was presented at a base frequency of *f* = 6 Hz. At every fifth position, an expressive face of the same individual (E) was displayed corresponding to a frequency of *f*/5 = 1.2 Hz (exemplary stimulation sequence: N N N N E N; see Figure 1). To avoid low‐level visual cue contamination, stimulus size varied randomly between 90% and 110% in 1% steps (e.g., 91% or 103%; Dzhelyova & Rossion, 2014).

In the max‐int task, participants saw a neutral face as base stimulus and a happy or angry expression (in separate blocks) at maximum intensity as deviant stimulus. Each emotion condition was presented twice, once with a male and a female identity, summing up to four stimulation sequences in two blocks. Each stimulation sequence had a duration of 40 s, resulting in a total length of 160 s of stimulation for the max‐int task.

In the grad‐int task, happy and angry expressions were shown in five different emotion intensity steps increasing from 20% to 100% in 20% increments (i.e., 20%, 40%, 60%, 80%, and 100%; Leleu et al., 2018). Each of the five intensity steps was displayed for 20 s in ascending order. Each emotion category block was shown twice with one male and one female identity, summing up to 20 stimulation sequences in 10 blocks with a total duration of 400 s.

To ensure participant's attention within the grad‐int task, we displayed a cartoon monkey face after every intensity step. Participants had to press a button whenever they saw the monkey's face. Two practice trials preceded the grad‐int task to familiarize children with the procedure. In both FPVS tasks, we reached a fixation rate of 90% (max‐int task: *M* = 91.1, *SD* = 10.6; grad‐int task: *M* = 89.3, *SD* = 6.2).

### 2.3 Emotion recognition task

Upon finishing the max‐int and grad‐int tasks, participants completed an explicit ERT (see Figure 6a). They saw a face morph stimulus from the grad‐int task on screen for a maximum of 3 s and had to indicate via a button press whether the face was neutral or yielding an emotion. Children had two buttons available for either neutral or expressive face decisions. Happy and angry faces were presented together with their associated neutral faces in separate blocks (60 trials each, 50 expressive trials [10 trials per intensity], and 10 neutral trials). Button order was randomized across participants; reaction times (RT) and accuracy rates were collected. Prior to the task, children were given five practice runs in order to acquaint themselves with the setup and the arrangement of buttons.

### 2.4 EEG acquisition and analysis

We collected continuous EEG with the QRefa Acquisition Software (Version 1.0 beta; MPI‐CBS, Leipzig, Germany) from 46 Ag/AgCl passive electrodes (EasyCap GmbH, Germany) while participants were seated in a cabin shielded against both sound and electromagnetic radiation. EEG data were sampled at 500 Hz (anti‐aliasing low‐pass filter of 135 Hz) and online referenced to CZ (ground electrode at Fp1). Electrode impedances were kept below 10 kΩ; electro‐oculograms were registered with electrodes at the outer canthi of both eyes and the orbital ridge of the right eye.

We carried out further offline pre‐processing in MATLAB R2016b using EEGlab (Delorme & Makeig, 2004). Data were high‐pass filtered at 0.01 Hz and low‐pass filtered at 100 Hz (IIR Butterworth filter, fourth order). To improve blink detection using independent component analysis (ICA; runica algorithm), we interpolated artifact‐ridden or noisy channels which showed artifacts through the recording (interpolation algorithm of EEGlab set to “spherical”; average channels across participants: max‐int: *M* = 1.5, *SD* = 0.9; grad‐int: *M* = 1.2, *SD* = 0.9). If artifacts (e.g., elicited through strong movement or sweating) were detected for a longer period of the trial, we excluded the participant from further analysis of the FPVS paradigm (as the number of trials was limited, this would have meant too much data loss, e.g., the max‐int task was comprised of two trials per condition). For each participant, components related to blink or saccade activity were automatically selected using the SASICA toolbox (Chaumon et al., 2015), manually re‐checked for accuracy, and subsequently removed (components on average max‐int: *M* = 3.5, *SD* = 1.3; grad‐int: *M* = 4.2, *SD* = 1.4). The data were re‐referenced to average reference.

#### 2.4.1 Frequency‐domain analysis

Each epoch was cropped to start at the onset of the first expressive face (max‐int: epoch length 40s [47 cycles]; grad‐int: 20 s [24 cycles]). Data were then averaged for the different emotions and tasks for each participant. Subsequently, a fast Fourier transform (FFT) was applied to every epoch with a high‐frequency resolution of 1/40 s = 0.0125 Hz for max‐int and 1/20s = 0.05 Hz for the grad‐int task.

Only frequencies corresponding to the frequency of the base rate or the expressive face and its harmonics were considered. (Harmonics are integer multiplies of a given frequency, i.e., for base response: 1 *f* = 6 Hz, 2 *f* = 12 Hz, 3 *f* = 18 Hz, etc.; for expression change response: 1 *f* = 1.2 Hz, 2 *f* = 2.4 Hz, 3 *f* = 3.6 Hz, etc.) To determine which of the responses carried meaningful signals, that is, were significantly different from noise, we first determined, on group‐level data, the range of harmonics to consider for further analyses with a *Z*‐score transformation: The FFT data were first averaged across all participants and conditions for each electrode; amplitudes at each harmonic of the base and expression change rate were extracted. The respective harmonic amplitudes at one frequency bin (*x*) were then baseline corrected and subsequently transformed into *Z*‐scores with the following formula (Lochy et al., 2019): *Z* = (*x*−mean noise amplitude)/(*SD* of the noise). For the max‐int task, the noise for each frequency bin was estimated from the respective surrounding 20 frequency bins with 10 bins on each side, excluding the immediately neighboring and the 2 most extreme values. For the grad‐int task, the number of bins was reduced to 10 frequency bins with 5 bins on each side (see Peykarjou, 2022: recommendation to use 10 bins for FPVS experiments with presentation times ≤30s). For each experiment, we determined *Z*‐scores >1.64 (or *p* < .05, one tailed).

Based on this criterion, for the max‐int task, we quantified significant average expression change responses by summing five harmonics: harmonics 1 (1.2 Hz) to 5 (7.2 Hz), excluding the harmonics corresponding to the base stimulation frequency (6 Hz). The base response was quantified as the sum of the response at the base rate (6 Hz) and two consecutive harmonics (12 and 18 Hz).

As in previous studies employing the grad‐int task (Leleu et al., 2018), the expression change (1.2 Hz) response was expected to be nearly absent at 20% of expression intensity and to increase with increasing intensity steps. Thus, in accordance with previous studies, we deviated from the *Z*‐score criterion (Dzhelyova et al., 2017; Leleu et al., 2018) and used the same harmonics for the extraction of the base and expression response as in the max‐int task.

**FIGURE 2:**
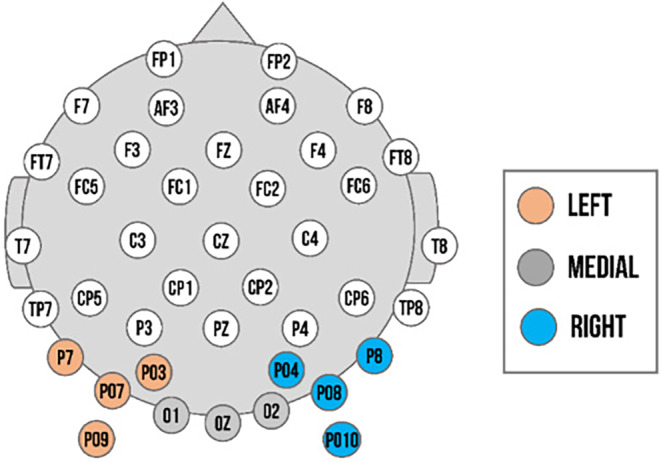
Electrode setup. Left cluster (electrodes: PO3, PO7, P7, and PO9) in orange; medial cluster (electrodes: O1, O2, and Oz) in gray; and right cluster (electrodes: PO4, PO8, P8, and PO10) in blue.

We determined our regions of interest in accordance with previous studies (Lochy et al., 2019; Vettori et al., 2019), confirmed by the greatest *Z*‐scores and visual inspection of the topographical maps of both groups (exact *Z*‐values available within data analysis material available online). For both experiments, the analysis of the base response and expression change response for the left cluster was comprised of PO3, PO7, P7, and PO9 in left occipito‐temporal area; the right cluster of PO4, PO8, P8, and PO10 in the right occipito‐temporal; and the medial cluster of O1, O2, and Oz in the medial occipital area (see Figure 2). We assessed the visibility of base responses for each individual and trial in the region of interest with the highest *Z*‐scores observed across participants. Only the data of participants who had observable baseline responses in these channels were utilized for the final analysis.

Two measures were employed to characterize responses in the frequency domain (Dzhelyova et al., 2017): baseline‐corrected amplitudes (BCAs) and SNRs. BCA values were calculated by applying a baseline correction to the FFT amplitude values. This was done by subtracting the mean amplitude of the surrounding noise at each frequency bin, using the same noise definition as above. As a final step, BCA values for significant harmonics (determined as described above) were summed to the final base and expression change responses. Summed BCA values were calculated on an individual level for each condition and region of interest (ROI) electrode. SNR values were computed for each participant and condition by dividing the FFT amplitude values at a given frequency bin by the mean amplitude of the estimated noise (same noise definition as above). SNRs were only used for visualization purposes and to describe the strength of the signal in relation to the noise.

In accordance with previous FPVS approaches (Leleu et al., 2018), we provided additional analyses including a physical dissimilarity index (PDI) to examine whether the significant amplitude increase in the expression change response as a function of intensity was not solely elicited by increasing physical changes between neutral and expressive faces. The PDI was obtained for each emotion with five linear steps for the increase in expression intensity. Afterward, summed BCAs were normalized for each participant, emotion, and intensity by dividing BCA values by the corresponding PDIs (detailed description in Leleu et al., 2018). These additional calculations were carried out for the expression change response analysis of the grad‐int task.

#### 2.4.2 Time‐domain analysis

Time‐domain analysis was performed to visualize the shape of the periodic changes time locked to the oddball stimulus and to estimate the speed and time course of facial expression change. We used a 30 Hz filter as well as an FFT notch filter to remove frequency information of the base frequency and its harmonics (Dzhelyova et al., 2017). By filtering out the base response and its harmonics from the EEG data, the signal provides direct expression change‐specific activities in the time domain. Data were segmented from −167 ms before stimulus onset to 667 ms post‐stimulus onset and baseline corrected using the mean activity during the 167 ms of the pre‐stimulus presentation. For the max‐int task, 90 trials per condition were available; for the grad‐int task, analyses contained 44 trials per intensity step. Segments containing artifacts were removed based on a semi‐automated artifact rejection of voltage (exceeding ±100 μV) and visual inspection of each trial. No differences between number of trials per facial expression were detected for max‐int (*t*(92) = −0.7, *p* = .5; happy: *M* = 76.2, *SD* = 10.3; angry: *M* = 74.7, *SD* = 11.2) or grad‐int (*t*(86) = 0.3, *p* = .8; happy: *M* = 35.9, *SD* = 4.0; angry: *M* = 36.1, *SD* = 4.3) after artifact rejection.

**FIGURE 3:**
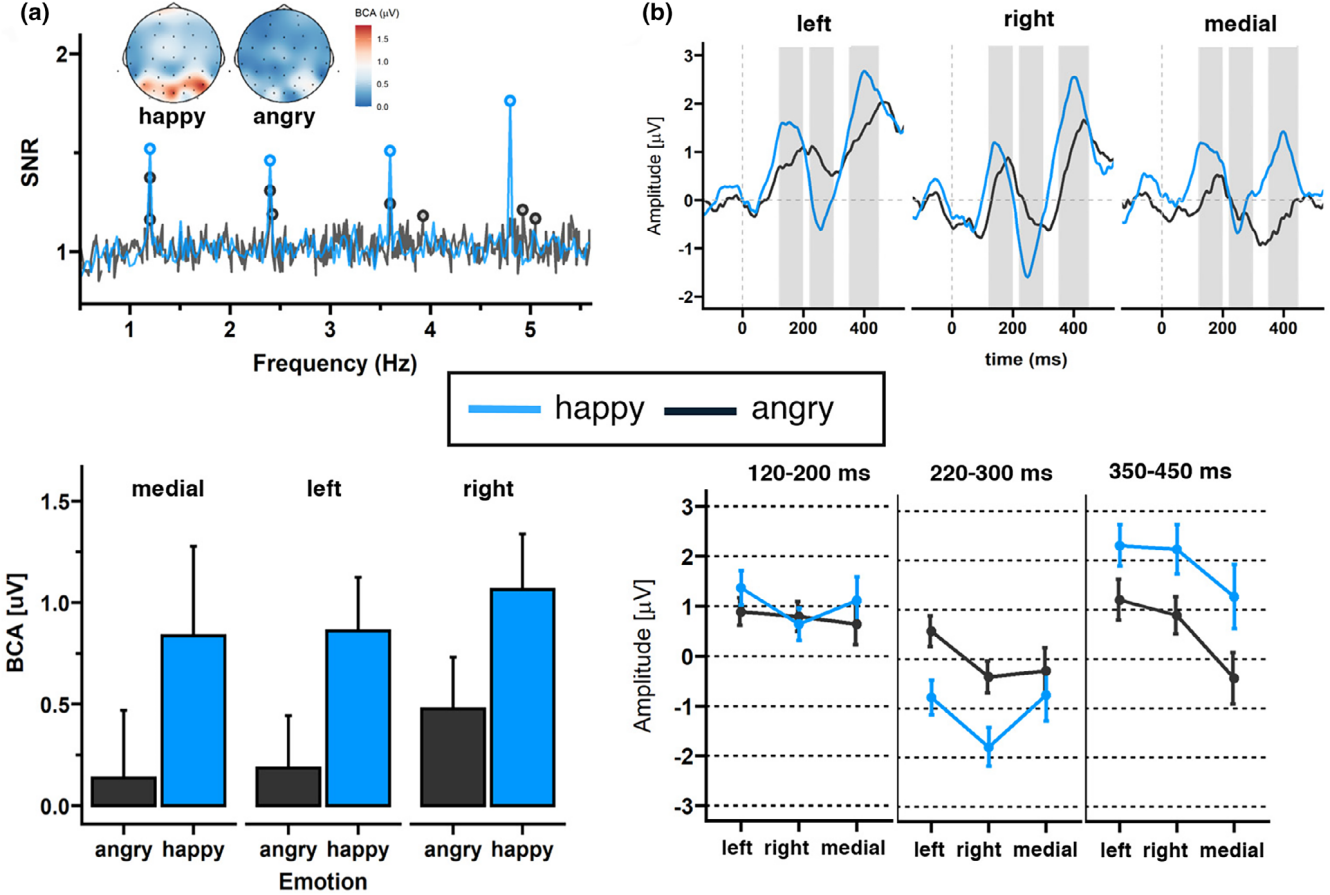
Visualization of max‐int task frequency‐ and time‐domain results. All results are separated for the different facial expressions (light blue = happy, dark blue = angry) and averaged across ROI channels (left cluster: PO3, PO7, P7, and PO9; right cluster: PO4, PO8, P8, and PO10; and medial cluster: O1, O2, and Oz). (a) Expression change response: (upper left panel) SNR spectra and scalp topographies (μV). (lower left panel) Bar graphs displaying BCA values. Error bars indicate standard errors (SE). (b) ERP waveforms and mean amplitudes: (upper right panel) Waveforms for C1, C2, and C3. Shadowed areas indicate the time windows used to identify participants' individual peaks and mean amplitudes. (lower right panel) Mean C1, C2, and C3 amplitudes and *SD*s separated for each time window.

**FIGURE 4:**
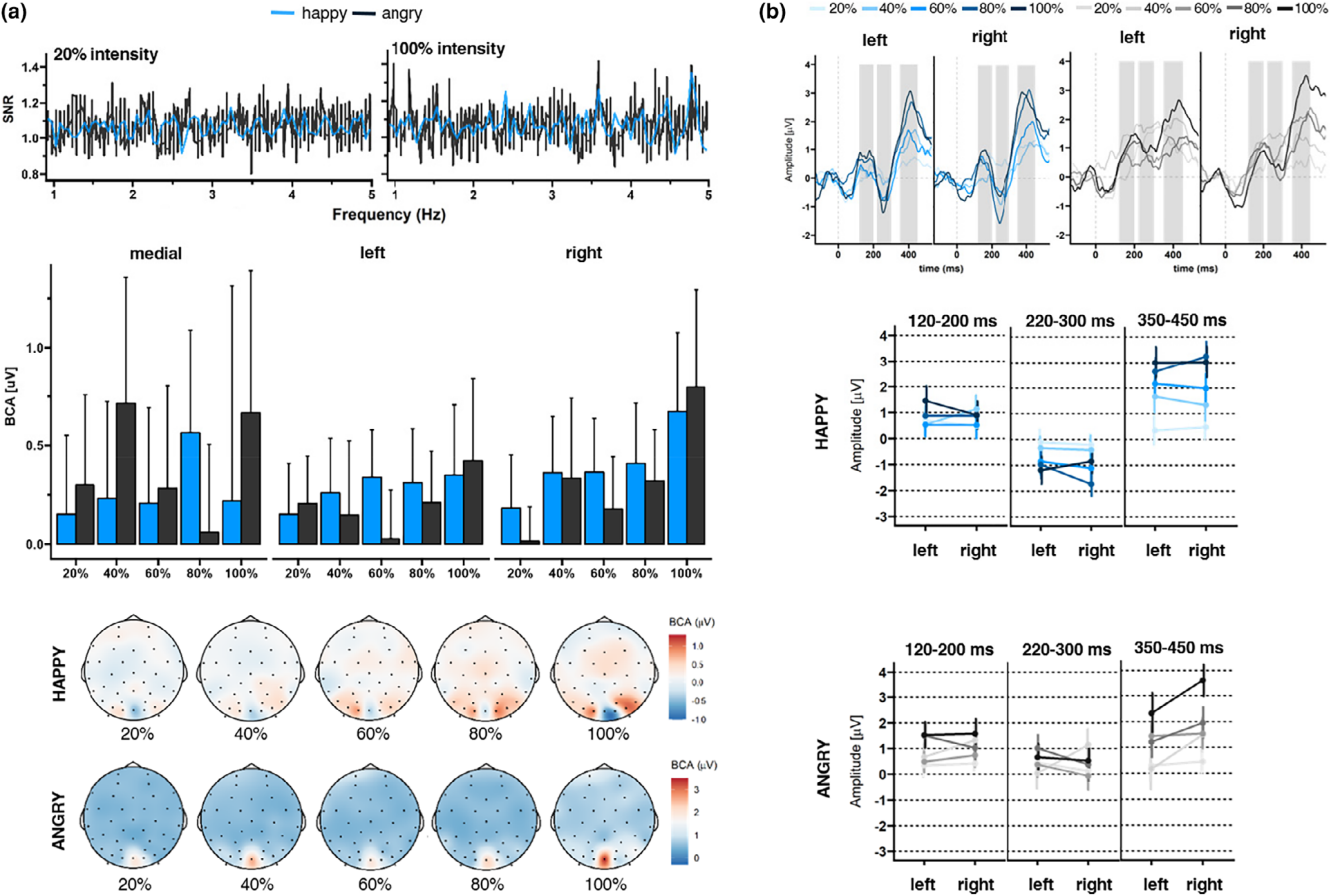
Visualization of the grad‐int task frequency‐ and time‐domain results. Happy and angry faces were presented with different intensities ranging from 20% to 100% intensity. All results are separated for the different facial expressions (light blue = happy and dark blue = angry, here also graded by intensity) and averaged across ROI channels (left cluster: PO3, PO7, P7, and PO9; right cluster: PO4, PO8, P8, and PO10; and medial cluster: O1, O2, and Oz). (a) SNR spectra for expression change response (upper left panels): [left panel] SNR values at 20% intensity. [right panel] SNR values at 100% intensity. (middle panel on the left) Bar graphs displaying BCA values. Error bars indicate standard errors (SE). (lower left panel) Scalp topographies (μV). (b) Waveforms and mean amplitudes: [upper right panels] Waveforms for C1, C2, and C3. Shadowed areas indicate the time windows used to identify participants' individual peaks and mean amplitudes. (lower right panels) Mean C1, C2, and C3 amplitudes and *SD*s are separated for each time window.

Regions of interest for the ERP components and time windows were based on previous research (Lochy et al., 2019; Vettori et al., 2019) and inspection of the ERP topographies averaged across all conditions and participants. For max‐int and grad‐int tasks, they matched with ROIs selected for the frequency domain analysis (left cluster: PO3, PO7, P7, and PO9; right cluster: PO4, PO8, P8, and PO10; and medial cluster: O1, O2, and Oz). In line with previous FPVS research (Leleu et al., 2018), a triphasic response reflecting the discrimination of an emotional expression from a neutral face was identified until approximately 500 ms after expression change onset (see Figures 3b and 4b). The three components (hereafter C1, C2, and C3, respectively, positive, negative, and positive) sequentially peaked at 176, 256, and 376 ms post‐expression change (time windows: C1: 120–200 ms, C2: 220–300 ms, and C3: 350–450 ms; comparable to Leleu et al., 2018). Peaks for each component were quantified as mean amplitude in a time window of 20 ms around the peak. During visual inspection of ERP topographies averaged across all conditions and participants, we did not detect the triphasic response pattern within the medial cluster in the grad‐int task. Thus, we only included data from the left and right clusters in the final analysis.

### 2.5 Statistical analysis

Statistical analyses were performed using R‐Studio (R Core Team, 2019). For all analyses, we conducted linear mixed model analyses using the packages lme4 (Bates et al., 2015) and sjstats (Lüdecke, 2021). For the analysis of the frequency domain in max‐int task, we examined BCAs with *emotion* (happy vs. angry expressions) and *ROI* (left vs. right vs. medial) as within‐subject factors and *group* (Zirkus Empathico vs. controls) as between‐subject factors. All analyses pertaining to the group variable can be found in Supplement S3. *Participant* was included as random intercept in the model. Models for the time‐domain analysis utilizing mean amplitudes as dependent variable were identical. We computed them separately for C1, C2, and C3. For the grad‐int task, we included *intensity* (20%, 40%, 60%, 80%, and 100%) as additional within‐subject factor in both frequency‐ and time‐domain analysis. We analyzed whether the BCA signal increased in response to the increase in expression intensity and identified the pattern that most accurately describes this increase (e.g., linear, cubic, or quadratic trend). Regarding the within‐subject factor ROI, we included the left and right clusters for the time‐domain analysis.

For the models of the RTs and accuracy rates of the ERT, we used the within‐subject factors emotion (happy vs. angry expressions) and intensity (20%, 40%, 60%, 80%, and 100%) as well as the between‐subject factor group (Zirkus Empathico vs. controls; analysis can be found in Supplement S3). Parallel to the other models, participant was included as random intercept.

Post hoc *t*‐tests were performed on the fitted model using the emmeans package (Lenth, 2019) with Tukey‐corrected *p*‐values used to compare means. We used partial eta‐squared (*η*_p_^2^) to quantify the size of effects (*η*_p_^2^; small effect: *η*_p_^2^ = .01, medium effect: *η*_p_^2^ = .06, and large effect: *η*_p_^2^ = .14; Keppel, 1991).

## 3 RESULTS

### 3.1 Max‐int: Dynamics of change for expression discrimination at maximal intensity

#### 3.1.1 Frequency domain: Base response and expression change response

A clear base response was visible for the base rate at 6 Hz response. The base reponse as well as the response of its harmonics, were characterized by a medial occipital topography peaking at channel O1 (*F* = 6 Hz: 1.01 μV, SNR = 5.63; 2F = 12 Hz: 0.59 μV, SNR = 5.77; and 3F = 18 Hz: 0.19 μV, SNR = 3.24). As shown in the upper left panel of Figure 3a, a clear expression change response was also visible. The highest SNR was detected at the fourth harmonic characterized by a right‐sided occipital topography peaking at P8 (F4 = 4.8 Hz: 1.19 μV, SNR = 1.75; Figure 3a upper panel).

Regarding the expression change response, the grand‐averaged summed BCAs for happy faces, pooled over participants and ROIs, revealed significant responses for changes from neutral to happy faces (0.92 μV ± 0.63, *z* = 2.61), but not from neutral to angry faces (0.28 μV ± 0.52, *z* = 1.50). SNR values for the 1.2 Hz response and corresponding harmonics were also numerically larger for happy versus angry faces.

Statistical analysis of the base response showed a significant main effect of emotion (*F*(2,225) = 6.10, *p* = .01, *η*_p_^2^ = .03; Figure 3a lower left panel), with happy faces eliciting a larger base response than angry faces. None of the other comparisons revealed significant results for the base response (see Table 2). For the expression change response, we also detected a significant effect of emotion (*F*(1,225) = 52.79, *p* < .001, *η*_p_^2^ = .18; Figure 3a lower panel), indicating larger BCA values for happy versus angry faces. Additionally, there was a main effect of ROI (*F*(2,225) = 4.17, *p* = .02, *η*_p_^2^ = .03), indicating larger BCA values for the right as compared to the medial cluster (*p* = .02). None of the other comparisons revealed significant results (see Table 2).

**TABLE 2:** Max‐int task frequency domain analysis: base and expression change response.

	Base response				Expression change response			
	df	*F*	*p*	*η*_p_^2^	df	*F*	*p*	*η*_p_^2^
Emotion	**225**	**6.09**	.**01**	.**026**	**225**	**52.79**	**<.001**	.**177**
ROI	225	1.32	.27	.011	**225**	**4.17**	.**02**	.**033**
Emotion × ROI	225	0.72	.49	.006	225	0.15	.86	.001

*Note:* Bold indicates significance level at *p* value < .05.

#### 3.1.2 Time domain: Amplitude analysis of ERPs

For C2, we found a significant emotion main effect (*F*(1, 77.769) = 43.47, *p* < .001, *η*_p_^2^ = .001), with happy faces eliciting a more negative amplitude than angry faces (see Figure 3b and Table 3). In addition, analyses revealed a significant ROI main effect (*F*(2, 778.001) = 8.80, *p* < .001, *η*_p_^2^ < .001). Amplitudes were largest in the right cluster compared to left (*p* < .001) or medial (*p* = .02) clusters. Regarding C3, we also detected significant main effects of emotion (*F*(1, 77.845) = 54.03, *p* < .001, *η*_p_^2^ = .001) and ROI (*F*(2, 77.801) = 15.47, *p* < .001, *η*_p_^2^ < .001). Happy faces elicited larger amplitudes than angry faces; amplitudes were lowest in the medial cluster compared to left (*p* < .001) and right (*p* < .001) clusters.

**TABLE 3:** Max‐int task: time‐domain analysis.

	Component 1				Component 2				Component 3			
	df	*F*	*p*	*η*_p_^2^	df	*F*	*p*	*η*_p_^2^	df	*F*	*p*	*η*_p_^2^
Emotion	77.815	2.38	.12	<.001	**77.769**	**43.48**	**<.001**	.**001**	**77.845**	**54.03**	**<.001**	**001**
ROI	77.801	1.57	.21	<.001	**77.801**	**8.80**	**<.001**	**<.001**	**77.800**	**15.47**	**<.001**	**<.001**
Emotion × ROI	77.801	1.94	.14	<.001	77.801	2.84	.05	<.001	77.800	0.44	.64	<.001

*Note:* Bold indicates significance level at *p* value < .05.

#### 3.1.3 Summary: Max‐int frequency‐ and time‐domain findings

We detected larger BCA values for both base and expression change responses as well as C2 and C3 amplitudes for happy versus angry faces. In both frequency and time domains, a dominance of the right hemisphere in processing expression change was observed.

### 3.2 Grad‐int: Dynamics of change for expression discrimination modulated by increasing intensities

#### 3.2.1 Frequency domain: Base response and expression change response

We detected a significant intensity main effect for the base response (*F*(4,1218) = 2.44, *p* = .04, *η*_p_^2^ = .01). Contrary to our expectations, the modulation of BCA values by intensity followed a cubic trend (*p* = .03), indicating response increases until 60%, followed by decreases in signal. In addition, we found a significant ROI main effect (*F*(2,1218) = 8.34, *p* < .001, *η*_p_^2^ = .01), with larger BCA values for the right versus left (*p* = .01) or right versus medial (*p* < .001) cluster. All statistical comparisons are reported in Table 4.

**TABLE 4:** Grad‐int task frequency domain analysis: base and expression change response.

	Base response				Expression change response			
	df	*F*	*p*	*η*_p_^2^	df	*F*	*p*	*η*_p_^2^
Emotion	1218	2.42	.12	<.001	1218	0.05	.82	<.001
Intensity	**1218**	**2.44**	.**04**	.**01**	**1218**	**2.86**	.**02**	.**01**
ROI	**1218**	**8.34**	**>.001**	.**01**	1218	1.74	.17	<.001
Emotion × Intensity	1218	0.53	.71	<.001	**1218**	**3.44**	.**01**	.**01**
Emotion × ROI	1218	0.31	.74	<.001	**1218**	**3.51**	.**03**	**<.001**
Intensity × ROI	1218	0.34	.95	<.001	1218	1.42	.18	.01
Emotion × Intensity × ROI	1218	0.37	.94	<.001	1218	1.77	.08	.01

*Note:* Bold indicates significance level at *p* value < .05.

Regarding the expression change response, the grand‐averaged summed BCAs for happy faces, pooled over participants and ROIs, revealed significant responses for changes from neutral to happy faces from 60% (60%: 0.28 μV ± 0.54, *z* = 2.05; 80%: 0.42 μV ± 0.58, *z* = 2.27; 100%: 0.31 μV ± 0.97, *z* = 2.16; see Supplement S4: Table S7) and neutral to angry faces from 80% (80%: 0.18 μV ± 0.46, *z* = 1.73; 100%: 0.62 μV ± 0.91, *z* = 2.24). In line with these observations, we did not detect identifiable SNR responses at 20% intensity (Figure 4a upper panel). In contrast, at 100% intensity, we found clearly identifiable SNR responses in particular for happy faces. Statistical analysis showed a significant intensity main effect (*F*(4,1218) = 2.86, *p* = .02, *η*_p_^2^ = .01), with larger BCA values for 100% versus 20% intensity (*p* < .001). The modulation of BCA values by intensity followed a linear (*p* < .01) and cubic trend (*p* = .04): Descriptively, a linear enhancement of BCA values was observed from 20% to 40% intensity and from 60% to 100% intensity. The cubic trend was observable between 40% and 100%, where a decrease in signal was visible between 40% and 60% followed by an increase up until 100% (see Figure 4a middle panel). In addition, there was a significant emotion × intensity interaction (*F*(4,1218) = 3.44, *p* = .01, *η*_p_^2^ = .01). However, within our additional analysis, in which we corrected with PDI values, the emotion × intensity interaction effect did not remain significant (*F*(4,1260) = 1.78, *p* = .13, *η*_p_^2^ = .01; see Supplement S5: Table S8 for statistical analysis with PDI correction). We also detected an emotion × ROI interaction (*F*(4,1218) = 3.51, *p* = .03, *η*_p_^2^ = .01), which survived PDI correction (*F*(4,1260) = 5.01, *p* = .01, *η*_p_^2^ = .01), as well as an emotion × intensity × ROI interaction (*F*(4,1260) = 2.53, *p* = .01, *η*_p_^2^ = .02). When separating analyses for each ROI, BCA values for the right cluster were larger for happy versus angry faces at 20% intensity (*p* = .01), whereas angry faces elicited larger BCA values than happy faces at 20% intensity in the medial cluster (*p* < .001; all other *p* > .05).

#### 3.2.2 Time domain: Amplitude analysis of ERPs

For C1, we detected a significant intensity main effect (linear trend: *p* < .001; all statistical parameters in Table 5).

**TABLE 5:** Grad‐int task: time‐domain analysis.

	Component 1				Component 2				Component 3			
	df	*F*	*p*	*η*_p_^2^	df	*F*	*p*	*η*_p_^2^	df	*F*	*p*	*η*_p_^2^
Emotion	124.877	3.05	.08	<.001	**124.875**	**112.58**	**<.01**	**<.001**	**124.869**	**16.56**	**<.01**	**<.001**
Intensity	**124.868**	**7.72**	**<.01**	**<.001**	**124.866**	**3.81**	**<.01**	**<.001**	**124.862**	**42.26**	**<.01**	**<.001**
ROI	124.846	0.06	.80	<.001	124.846	1.24	.26	<.001	**124.846**	**6.93**	.**01**	**<.001**
Emotion × Intensity	124.873	1.05	.38	<.001	**124.872**	**6.16**	**<.01**	**<.001**	**124.867**	**3.66**	.**01**	**<.001**
Emotion × ROI	124.846	0.19	.66	<.001	124.846	0.01	.92	<.001	**124.846**	**5.31**	.**02**	**<.001**
Intensity × ROI	124.846	1.33	.25	<.001	124.846	2.21	.06	<.001	124.846	1.02	.39	<.001
Emotion × Intensity × ROI	124.846	0.71	.58	<.001	124.846	1.22	.30	<.001	124.846	1.32	.26	<.001

*Note:* Bold indicates significance level at *p* value < .05.

For C2 (see Figure 4b), we detected significant main effects of emotion and intensity (linear trend: *p* < .01), which where qualified by an emotion × intensity interaction. Except for 20% intensity (*p* = .09), happy faces elicited larger amplitudes than angry faces across intensities (all *p* < .001).

For C3, we found significant main effects of emotion (happy > angry), intensity (linear trend: *p* < .001), as well as ROI (right cluster > left cluster). As shown in Table 5, several two‐way interactions were significant, which were qualified by two three‐way interactions (emotion × intensity × group and emotion × group × ROI).

#### 3.2.3 Summary: Grad‐int frequency‐ and time‐domain findings

Regarding the frequency domain, we detected a cubic modulation by intensity for the base response, and linear as well as cubic trends for the expression change response. At 20% intensity, larger BCA values for happy versus angry faces were observed in the right cluster, whereas the opposite pattern was detected in the medial cluster.

With regard to the time domain, C2 (perceptual coding) and C3 (emotion categorization) indicated an increase in signal with increasing intensity as well as larger amplitudes for happy versus angry faces.

### 3.3 Exploratory analysis: Individual neural trajectories for facial expression detection

**FIGURE 5:**
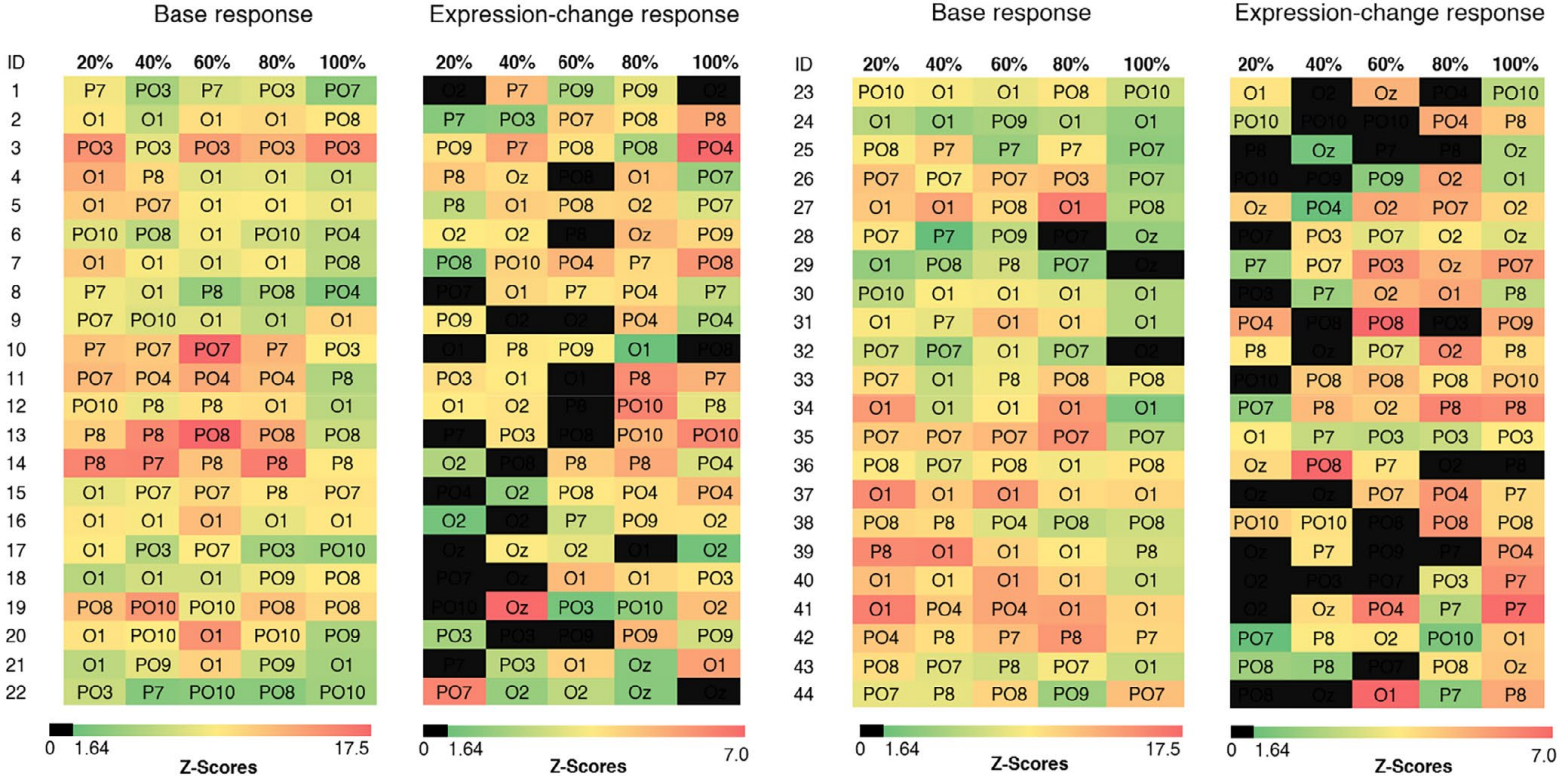
Color‐coded table depicting the strength of the greatest *Z*‐scores (and its corresponding channel) at each intensity step averaged, separated for base and expression change response across emotions for each individual participant.

We determined each participant's neural threshold for significant base and expression change responses. Given that after PDI correction intensity did not interact with emotion (although both happy and angry faces did elicit significant responses), individual responses were averaged across emotions and their significance was estimated using the same criterion as for the group‐level analysis (see Figure 5).

Regarding the base response, the cubic trend detected in the statistical analysis was visible: When visually inspecting the data, we observed signal increases up until 60% intensity which were then followed by decreases or fluctuations (e.g., significant response for 80%, but not 100% intensity). For significant expression change responses, we observed an increase in participants for increasing expression intensity for both happy (at 20% intensity: 48% of participants, at 40%: 43%, at 60%: 52%, at 80%: 66%, and at 100%: 66%) and angry faces (at 20% intensity: 36% of participants, at 40%: 30%, at 60%: 43%, at 80%: 55%, and at 100%: 61%). When calculating a linear mixed model similar to the one of the grad‐int task in the main analysis (without the variables ROI and group), we detected main effects for emotion (*F*(1,387) = 4.51, *p* = .034, *η*_p_^2^ = .01), with confirmed expression change rates for happy being larger than for angry faces and intensity (*F*(4,387) = 4.74, *p* < .001, *η*_p_^2^ = .04). Post hoc tests indicated a significant increase between 20% and 100% (*p* = .1, all other *p* > .05). We detected a clear linear trend (*p* > .001, in comparison: quadratic trend: *p* = .21 and cubic trend: *p* = .36), suggesting a linear increase in expression change response with increasing expression intensity. Topographically, response patterns were quite variable, with many participants showing their maximum values at 80/100% intensity at electrodes PO4 (23%) and P8 (19%) both located within the right cluster.

### 3.4 Behavioral measure: Explicit facial emotion processing

**FIGURE 6:**
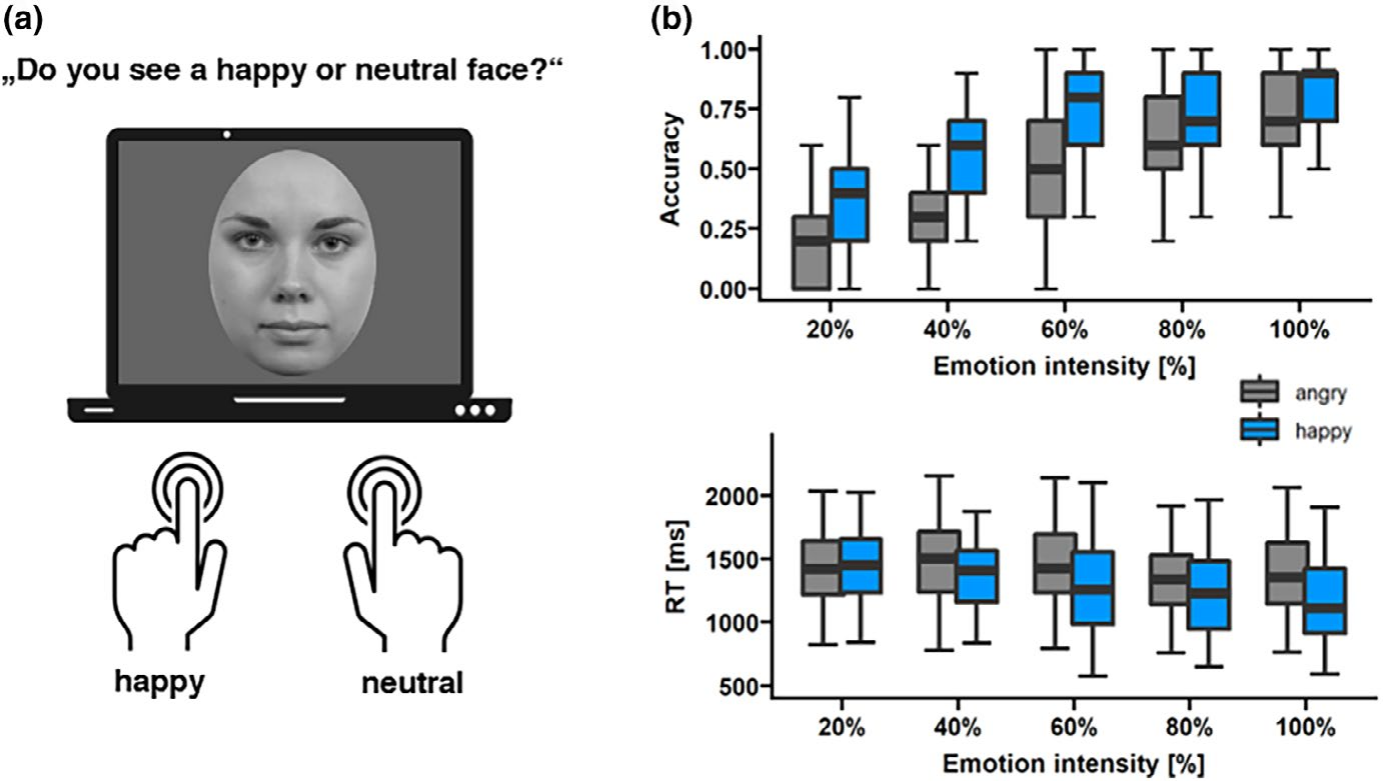
Emotion recognition task (ERT). (a) Display of task. (b) Accuracy findings (upper panel) and reaction time findings (lower panel).

As shown in Figure 6b (upper panel), we detected that emotion (χ^2^ (1, *N* = 44) = 104.27, *p* < .001) had a significant effect on accuracy rates, with happy faces being detected more accurately than angry faces. In addition, there was a significant intensity main effect (χ^2^ (1, *N* = 44) = 104.27, *p* < .001), indicating that accuracy increased with increasing expression intensity. This relationship was a mixture of linear (*p* < .001) and quadratic components (*p* < .001).

Regarding reaction times visualized in Figure 6b (lower panel), we detected significant main effects of emotion (*F*(1,10) = 38.8, *p* < .001, *η*_p_^2^ = .007) and intensity (*F*(4,10) = 7.95, *p* = .004, *η*_p_^2^ = .005), which was also qualified by an emotion × intensity interaction (*F*(4,10) = 3.97, *p* < .03, *η*_p_^2^ = .003). Happy faces were detected faster than angry faces at 60% (*p* < .001), 80% (*p* < .001), and 100% (*p* < .001) intensity. The relationship between reaction time and intensity was characterized by a linear trend (*p* = .001).

## 4 DISCUSSION

Whereas previous ERP research focused on the neural patterns of processing different facial expressions, we investigated an implicit measure of preschoolers' ability to detect brief changes in facial expressions (happy and angry expressions), employing the FPVS approach. We used two FPVS tasks: Within the first task, we investigated whether preschoolers detect brief changes when emotional facial expressions are presented at maximal intensity (max‐int task). The second task included face morphs gradually increasing in expression intensity to inquire which threshold is needed to detect a facial expression change (grad‐int task).

Within the max‐int task, we found reliable expression change responses for happy and angry faces within the frequency and time domain. In addition, we also detected larger expression change rates for happy versus angry faces. When examining the gradual increase in emotion intensity, we detected a linear increase in expression change responses, whereas the trajectory of the base response followed a cubic trend. In line with the max‐int task, differences in expression change responses were apparent within SNRs as well as later components in the time domain indicating larger expression change responses for happy in comparison to angry faces. Max‐ and grad‐int task findings are paralleled by higher accuracy rates and faster RTs within the ERT, indicating a processing advantage of happy over angry faces in our preschool sample. The analysis of individual trajectories revealed large inter‐individual differences, with the majority of the sample showing significant expression change responses at 60% intensity.

Our first research question aimed to investigate whether brief changes in expression can be detected in preschoolers' brain responses. In line with previous FPVS research (Dzhelyova et al., 2017; Leleu et al., 2018), significant expression change responses within the max‐int and grad‐int tasks provide evidence for this hypothesis. The results highlight the value of the FPVS approach to address further developmental questions as it offers an implicit, fast, and objective way to measure the ability to detect expression change. Although the focus of our research was on preschoolers with typical development, the encouraging outcomes also indicate potential applicability to clinical populations that exhibit unique emotional processing challenges, such as individuals on the autism spectrum. Due to its concise and implicit characteristics, FPVS may be especially well suited for individuals who lack verbal communication abilities or have a limited attention span. In fact, other studies have already successfully used the FPVS approach in the context of individual face (Vettori et al., 2019, 2020) and facial expression discrimination (van der Donck et al., 2019) in autistic 8‐ to 12‐year‐old boys.

Regarding our second research aim, we examined differences in expression change for different emotions. In line with our hypothesis, we detected larger responses for happy versus angry faces, which was particularly visible in the max‐int task. This finding is paralleled by previous behavioral research (Durand et al., 2007; Gao & Maurer, 2010) as well as our ERT findings, which indicated a processing advantage of happy faces. In addition, as part of the pre‐registered training study, we also conducted a passive face viewing task to measure ERPs, in which we also detected larger activity for happy faces in components P1 and P3 (Naumann et al., 2023). Thus, we would conclude that happy faces are more readily processed than angry faces by preschoolers, providing further support for the framework of the progressive development of emotions (Camras & Halberstadt, 2017). The slower development of angry or other emotions with a negative valence such as fear or sadness could be explained by the generally lower exposure to these emotions in daily lives of preschoolers (Gao & Maurer, 2010; Leppänen et al., 2007). Furthermore, previous research formulated an initial negativity hypothesis, assuming that a preliminary bias may exist toward processing emotionally ambiguous faces, such as neutral expressions with a negative valence (Petro et al., 2018; Rollins et al., 2021). Particularly children under the age of 9 seem to interpret neutral faces ambiguously (either more positive or negative; Durand et al., 2007). Taken together with the fact that changes in facial expression were presented very briefly (under 200 ms), these factors might have contributed to the lower expression change responses for angry faces.

Unexpectedly, we also identified modulations by emotion in the base response, which may indicate heightened attention to positive emotions. The finding is consistent with previous FPVS research, which proposed that the base response could potentially represent visual processes at both low and high levels (Dzhelyova et al., 2017; Dzhelyova & Rossion, 2014). It also aligns with prior more general frequency tagging research demonstrating that SSVEPs were modulated by attention, specifically exhibiting increased amplitudes when participants concentrated on a task as compared to when they had to ignore it (Toffanin et al., 2009). Based on an examination of the signals' topography of our max‐int task, the base responses for happy over angry faces seem to be larger in the left and right clusters. Previous FPVS research has indicated that this pattern is more indicative of high‐level visual responses (Dzhelyova et al., 2017; Liu‐Shuang et al., 2014). Thus, attentional and visual processes may have influenced the base response of the max‐int task. It appears imperative to examine the extent to which the FPVS task responses are in fact linked to affective processing, as opposed to processing that is solely perceptual. When considering individual facial characteristics, a happy face generally comprises a smile. Research has demonstrated that this facial feature is more conspicuous than any other facial area on happy or unhappy faces (Calvo & Nummenmaa, 2008). This is further corroborated by the smile's luminance (e.g. as teeth are visible), contrast, and spatial orientation (Borji & Itti, 2013). Hence, being more salient and distinctive may have impacted the modulation of the change response for happy faces. We controlled for the physical properties of our stimuli and used a PDI (Leleu et al., 2018) to control for contrast differences. The possibility that expression recognition may have mainly relied on the perceptual analysis of visual features rather than emotional meaning, however, cannot be clearly excluded (Calvo & Nummenmaa, 2016), particularly because of the brief changes of expression. However, one could argue that this highly controlled decontextualized experimental setting can only be seen as first proxy to real‐life behavior and that, in daily life, faces appear in context where the affective evaluation plays a detrimental role (Calvo & Nummenmaa, 2016).

For the grad‐int task, there was no significant difference between emotions, which is in line with previous research using a similar research design for adults (Leleu et al., 2018). As the stimulation sequence duration was halved compared to the max‐int task, one could argue that effects might have been hampered by lower statistical power. In addition, particularly in the conditions with low expression intensities, we barely detected any expression change responses (Leleu et al., 2018). Thus, it is possible that, due to this reduced power, effects were less likely to be detected. However, differences were visible within the time‐domain analysis of the grad‐int task, displaying larger amplitudes for happy versus angry faces at C2 and C3 connected to perceptual encoding and emotion categorization (Leleu et al., 2018). This finding can be related to previous ERP research in preschoolers, where modulations by emotion were found for the higher‐order P3 component (Naumann et al., 2022, 2023).

As our third research aim, we investigated the intensity thresholds for the detection of expression change. Confirming previous research (Leleu et al., 2018), we found an increase in expression change response with increasing expression intensity. When analyzing the trend for the base response, however, we did not find an adaption or decrease in signal (Leleu et al., 2018), but a cubic trend. Relating to the individual trajectories of base and expression change responses, we detected that up until 60% of expression intensity, the neutral face may not be well distinguishable from the expressive face within this age range. This finding aligns with prior behavioral research indicating that preschoolers require a minimum of 40% intensity of emotion for recognition (Rodger et al., 2018, 2015). Furthermore, research showed that different age groups employ distinct strategies and neural structures for face recognition (Taylor et al., 2011), potentially contributing to the observed differences in neural patterns. Our task also slightly diverted from the design of Leleu et al. (2018) in that we added short breaks between each intensity step. This procedure allowed us to provide children better opportunities to take breaks, which in turn reduced movement artifacts and enhanced compliance. As we detected the same linear trend for the expression change response as in Leleu et al. (2018), we would see this diversion from the previous protocol as less likely to have modulated the responses. Lastly, the large variability in thresholds could also in part be caused by general factors such as skull thickness and cortical folding (Vettori et al., 2020).

Even though the FPVS tasks were included as part of larger training study comparing the digital training Zirkus Empathico to a control training (Naumann et al., 2023), we focused on the neural mechanisms of facial expression processing in preschoolers and implemented the factor training as a control variable. Both in maximum and gradual intensity, some analyses showed differences in neural response between the Zirkus Empathico and the control group (see Supplement S3: Tables S3–S6 for detailed results). However, findings were inconclusive and require careful interpretation: Within the max‐int task, we found larger C3 amplitudes for happy faces in the Zirkus Empathico group compared to controls. This result is similar to the ERP task results obtained during the training study (Naumann et al., 2023), which demonstrated greater P3 amplitudes for happy faces compared to neutral and angry faces within the Zirkus Empathico group. Thus, we derive the hypothesis that training differences may be detectable in later processing stages previously associated with the in‐depth analysis of emotion (Naumann et al., 2022; Schindler & Bublatzky, 2020). However, these results represent only a fraction of frequency‐ and time‐domain results, whereas in most of the other analyses, we detected null results for the modulation of training. Furthermore, a post hoc power analysis revealed a low achieved power for both the max‐int and grad‐int tasks regarding the expression change response analyses of the training effects (max‐int task: 15%; grad‐int task: 12%). Additionally, a substantial number of participants would be required to identify a true effect in the data. Vettori et al. (2019) found large effects for group differences in their expression change response, but they compared differences in children with and without autism. Our sample was comprised of children without any clinical history being more homogenous from the beginning, which may have led to smaller training effects in the end. The study therefore serves as a starting point for using FPVS to disentangle training effects in young samples displaying implications for needed sample sizes for non‐clinical groups and modification to the FPVS paradigm (e.g., number of FPVS variants or trials).

Overall, the FPVS results align with previous ERP findings (Naumann et al., 2023), offering first evidence that FPVS and ERP exhibit comparable sensitivity to grasp facial expression processing in preschoolers. The FPVS approach, however, seems to show clear advantages compared to the ERP method regarding execution speed and robustness, even at the individual level. In addition, FPVS data can be analyzed in the frequency and time domain, allowing for a better comprehension of synchronization and temporal brain processes.

As for the study's limitations, we examined data from a controlled, narrow‐aged preschool sample which allowed us to learn more about the developmental processes of this specific age range. However, we cannot derive conclusions for other age groups as development unfolds rapidly within the first years of life (Denham, 2018; Denham et al., 2009). Our sample also consisted of families of European origin who had a middle‐to‐high socio‐economic status, which further limits generalizability.

Future research may benefit from incorporating various methods to quantify EEG signals, such as ERPs and frequency band analyses, to elucidate neural similarities and differences that can be mapped using these diverse approaches. FPVS could also be employed cross‐sectionally from young childhood to early adulthood to understand how frequency markers evolve across development. Particularly analyses of individual thresholds for detecting facial expressions could be of value to elaborate which emotion intensity is needed to be reliably detected by the brain. As the FPVS approach due to its speed and implicit nature offers ways to overcome the limited attention span or non‐verbality of participants, it could be expanded to clinical groups with these restrictions who show difficulties in emotion processing (e.g., depression and social anxiety). In our research, we found a first indication that expression change responses differ regarding the emotional valence of the faces. The distinction and interaction between affective and other perceptual differences have yet to be fully determined. Thus, it would be of interest as well to integrate more basic and complex emotions (Calvo & Nummenmaa, 2016) to detect whether they have distinct FPVS patterns for each emotion. Considering the variability of the smile on a happy face, it would also be beneficial to ascertain whether our findings can be extrapolated to distinct varieties of happy faces (e.g., with or without teeth). In addition, our study used highly controlled as well as high‐intensity facial expressions from a limited number of facial identities. Future efforts could test whether differences in expression change response endure in the presence of more naturalistic (e.g., more subtle) emotional expressions. Furthermore, effects should be investigated by incorporating a greater variety of facial identities, for instance, in order to discern processing variations based on age and gender (Schneider et al., 2022). The variation of both face identities in the base and expression change responses is essential, as stable effects would enhance the task's validity and generalizability. Moreover, greater trial diversity enhances the probability that young participants remain interested and attentive, resulting in overall increased task compliance. To investigate whether differences in expression response are linked to an actual change in facial expression or solely alterations in facial features, an experimental condition could be added in which the deviant stimulus contains perceptual differences devoid of emotional connotations (e.g., sequence of neutral faces interleaved with a neutral face depicting an open mouth). Furthermore, to complement this neural measure, more behavioral aspects of emotion knowledge or broader socio‐emotional competence could be included to understand the strength of association between this neural marker and observable behavior.

## 5 CONCLUSION

In this initial investigation of emotion perception using FPVS in preschoolers, we identified a consistent index of expression change, demonstrating enhanced processing for happy faces relative to angry faces. The findings highlight the potential of the FPVS approach to address further developmental questions as it offers an opportunity to measure the ability to detect expression change implicitly, quickly, and objectively.

## AUTHOR CONTRIBUTIONS

**Sandra Naumann:** Conceptualization; data curation; formal analysis; funding acquisition; investigation; methodology; project administration; visualization; writing – original draft; writing – review and editing. **Mareike Bayer:** Conceptualization; methodology; resources; supervision; validation; writing – review and editing. **Isabel Dziobek:** Conceptualization; funding acquisition; resources; software; supervision; writing – review and editing.

## FUNDING INFORMATION

This study was supported by funding from the Berlin School of Mind and Brain, Humboldt‐Universität zu Berlin, and the Stiftung der Deutschen Wirtschaft (sdw).

## CONFLICT OF INTEREST STATEMENT

The authors declare no conflicts of interest.

## Supporting information


Data S1.

## Data Availability

Data and code necessary to reproduce all analyses reported here and additional Supplementary Files are available at https://osf.io/8vuwt/.
