# Supplementary material for: Enhanced neural sensitivity to brief changes of happy over angry facial expressions in preschoolers: A fast periodic visual stimulation study
Source: Psychophysiology. 2024 Nov 18;62(1):e14725. doi: 10.1111/psyp.14725 (PMC11775875; doi:10.1111/psyp.14725)
Supplement: Supplementary file 1 — Data S1. [file PSYP-62-e14725-s001.pdf]

Enhanced neural sensitivity to brief changes of happy over angry facial expression in preschoolers: A fast periodic visual stimulation study

Sandra Naumann[1, 2, *] | Mareike Bayer[1, 2] | Isabel Dziobek[1, 2]

[1] Berlin School of Mind and Brain, Humboldt-Universität zu Berlin, Berlin, Germany
[2] Department of Psychology, Institute of Life Sciences, Humboldt-Universität zu Berlin, Berlin, Germany
* Correspondence: sandra.naumann@hu-berlin.de

Supplementary material

DESCRIPTION S1 ..... **2**
ADDITIONAL DEMOGRAPHICS S2 ..... **4**
ADDITIONAL ANALYSIS S3 ..... **5**
ADDITIONAL ANALYSIS S4 ..... **7**
ADDITIONAL ANALYSIS S5 ..... **8**

Data availability: Data and code necessary to reproduce all analyses reported here and additional supplementary files are available at https://osf.io/8vuwt/ .

DESCRIPTION S1: Description of training study

Study overview

The study protocol was pre-registered at the German register for clinical studies (DRKS-ID: DRKS00015789). It included a training group which received the socio-emotional touchscreen application Zirkus Empathico (Kirst et al., 2015; Kirst et al., 2022) and an active control group which interacted with the language learning application Squirell and Bär (the Good Evil GmbH). Eligible participants were randomly allocated to the Zirkus Empathico or control group. Baseline socio-emotional competence (targeting empathy, emotion recognition, prosocial behavior) was examined with child assessments and parent ratings prior to training assignment at the study center. For the first measurement, no EEG data was collected. Each family then received a tablet-PC with the assigned touchscreen application to practice at home. Training lasted six weeks, with a weekly engagement of minimum 60 minutes. After six weeks of training at home, parent ratings and child assessments were repeated at the study center by an evaluator who was blind to group assignment. Furthermore, children participated in an EEG measurement. Within the EEG assessment, the maximum-intensity (max-int) and gradual intensity (grad-int) fast periodic visual stimulation (FPVS) task were administered.

Description of training and control touchscreen applications

Zirkus Empathico targets awareness and differentiation of own and others' emotions, empathy, and prosocial behaviors through interactions with naturalistic video sequences of facial expressions and social situations (Kirst et al., 2015; Kirst et al., 2022). The touchscreen application consists of 4 modules and an emotion library. In the first module, the child gets to know the virtual emotion manikin for the first time. The manikin constitutes a central element of the training to support the child in expressing perceived emotions on a two-dimensional scale indicating arousal and valence levels. Firstly, the child can specify its inner emotional state regarding a specific context (emotion-inducing video clip; see Figure 1). In a second step, the child describes its inner state by choosing an emotion label. Within the second module, the child is asked to identify emotion labels for facial expressions of adult and child protagonists (emotions: happy, sad, angry, anxious, and surprised). The third module requires the child to identify a specific emotion-eliciting context of another person. The child chooses the correct emotion label from 3 options. Within the last module, the child is presented with a third person's emotional expression embedded in an emotion-triggering context. Afterward, the child can decide how to react to the other person (e.g., go and talk to this person), fostering empathy and prosocial actions. Lastly, the library contains explanations of the emotion manikin and the six emotion cards with definitions and explanations of the basic emotional states targeted.

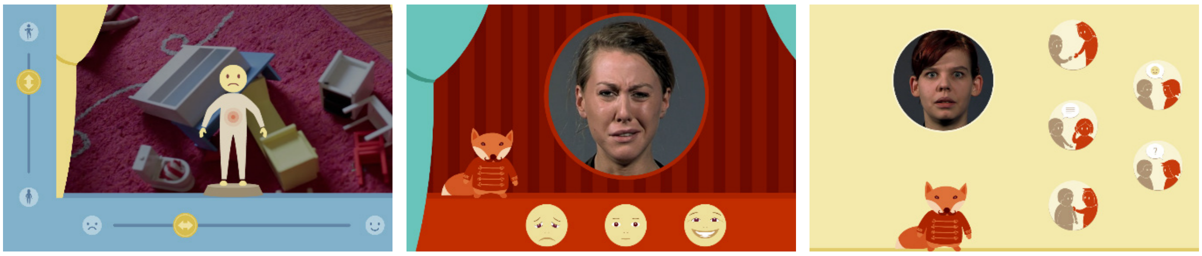

Figure 1. Training elements of Zirkus Empathico. Left: Module 1 - Understanding own emotions with the virtual manikin. Middle: Module 2 – Detecting and understanding others' emotions . Right: Module 4 – Learning how to react to others' emotions.

As control training, we employed the touchscreen application Squirell & Bär (the Good Evil GmbH), which fosters early foreign language acquisition through the interaction with basic English words and phrases. The child follows a story about a squirrel and a bear who are given the task of saving the bees from extinction. The children learn basic words (e.g. names of animals, food, etc.) as well as first ways to start a conversation (e.g. introduce themselves, ask for directions).

ADDITIONAL DEMOGRAPHICS S2: Participant characteristics per group for each FPVS condition

Table 1. Participant characteristics per group for max-int task

Characteristics		controls (N = 22)	Zirkus Empathico (N = 25)
Sex	Female / Male	10 / 12	11 / 14
Age	Years *M (SD)*	5.2 (0.9)	5.4 (0.9)
SES (Winkler Index)	Low *n* (%)	2 (9)	2 (8)
	Medium *n* (%)	20 (91)	22 (88)
	High *n* (%)	0 (0)	1 (4)
Verbal age	PPVT percentiles *M (SD)*	67.4 (25.7)	62.0 (29.0)
Nonverbal IQ	CPM score *M (SD)*	15.2 (4.0)	15.0 (4.1)

SES = socioeconomic status (Winkler Index; Winkler & Stolzenberg, 1998), PPVT = Peabody Picture Vocabulary Test, CPM = Coloured Progressive Matrices.

Table 2. Participant characteristics per group for grad-int task

Characteristics		controls (N = 22)	Zirkus Empathico (N = 22)
Sex	Female / Male	12 / 10	10 / 12
Age	Years *M (SD)*	5.2 (0.9)	5.4 (0.9)
SES (Winkler Index)	Low *n* (%)	2 (9)	1 (5)
	Medium *n* (%)	20 (91)	19 (86)
	High *n* (%)	0 (0)	2 (9)
Verbal age	PPVT percentiles *M (SD)*	69.1 (25.0)	60.9 (29.2)
Nonverbal IQ	CPM score *M (SD)*	15.5 (4.0)	15.0 (4.4)

SES = socioeconomic status (Winkler Index; Winkler & Stolzenberg, 1998), PPVT = Peabody Picture Vocabulary Test, CPM = Coloured Progressive Matrices.

ADDITIONAL ANALYSIS S3: Processing differences for controls vs. Zirkus Empathico group

Group differences for the max-int task

Frequency domain

No group differences were detected within the frequency domain analysis (complete statistical information in Table 3).

Time-domain

For C1, we detected a significant group x ROI interaction ($F(2, 77801) = 3.86, p = .02, \eta_p^2 < .001$; complete statistical information in Table 4). Compared to controls, participants of the Zirkus Empathico group showed larger amplitudes in the right cluster (p = 04). For C3, we found a group x ROI interaction ($F(1, 77801) = 3.18, p = .04, \eta_p^2 < .001$), which was further qualified by an Emotion x Group x ROI interaction ($F(2, 77801) = 3.33, p = .04, \eta_p^2 < .001$). Post-hoc tests indicated enhanced amplitudes for happy faces in the lOT cluster for the Zirkus Empathico group as compared to controls ($p = .02$).

Group differences for the grad-int task

Frequency domain

Within the base response, we detected a significant group x ROI interaction ($F(2,1218) = 3.77, p = .02, \eta_p^2 = .01$), however, none of the post-hoc tests were significant (all $p > .05$). Regarding the expression change response, we detected a significant intensity x group interaction ($F(2,1218) = 3.49, p = .01, \eta_p^2 = .01$; complete statistical information in Table 5). Post-hoc tests implied that controls exhibited larger expression change responses than the Zirkus Empathico group at 100% intensity ($p < .001$). None of the post-hoc tests were statistically significant (all $p > .13$).

Time domain

For C1, we found a four-way interaction of emotion x intensity x group x ROI ($F(4,124846) = 2.99, p < .02, \eta_p^2 < .001$). Therefore, we examined left and right clusters separately to simplify analyses. Only within the left cluster, we detected another significant effect, namely an emotion x intensity x group interaction ($F(4,62425) = 3.96, p < .01, \eta_p^2 < .001$). At 100% intensity, controls showed larger amplitudes for angry faces compared to the Zirkus Empathico group (all other post-hoc tests: $p > .08$).

For C2, the significant intensity x group interaction did not contain any significant post-hoc test results (all $p < .05$). We found a significant emotion x group interaction, with both groups showing larger values for happy vs. angry faces. Lastly, we detected an intensity x group x ROI interaction. Compared to controls, the Zirkus Empathico group showed enhanced amplitudes in the right cluster at 60% intensity ($p < .001$).

For the emotion x intensity x group interaction at C3, post-hoc tests revealed that amplitudes

for angry faces at 100% intensity were larger for controls vs. Zirkus Empathico participants (*p* = .03). The other three-way interaction did not reveal any significant post-hoc results (all *p* > .1).

Table 3. Max-int task frequency domain analysis: Base and expression change response.

	**Base response**				**Expression change response**			
	df	*F*	*p*	η_p^2	df	*F*	*p*	η_p^2
Group	45	0.06	.81	< .001	45	0.83	.37	.003
Emotion	**225**	**6.09**	**.01**	**.026**	**225**	**52.79**	**< .001**	**.177**
ROI	225	1.32	.27	.011	**225**	**4.17**	**.02**	**.033**
Group x Emotion	225	0.34	.56	.001	225	0.03	.87	< .001
Group x ROI	225	0.14	.87	.001	225	3.12	.05	.025
Emotion x ROI	225	0.72	.49	.006	225	0.15	.86	.001
Group x Emotion x ROI	225	0.40	.67	.003	225	0.01	.99	< .001

Table 4. Max-int task: Time domain analysis

	**Component 1**				**Component 2**				**Component 3**			
	df	*F*	*p*	η_p^2	df	*F*	*p*	η_p^2	df	*F*	*p*	η_p^2
Group	45	0.53	.47	< .001	45	0.54	.47	< .001	45	1.21	.28	< .001
Emotion	77815	2.38	.12	< .001	**77769**	**43.48**	**< .001**	**.001**	**77845**	**54.03**	**< .001**	**001**
ROI	77801	1.57	.21	< .001	**77801**	**8.80**	**< .001**	**< .001**	**77800**	**15.47**	**< .001**	**< .001**
Group x Emotion	77815	2.00	.16	< .001	77769	2.81	.09	< .001	77845	1.30	.25	< .001
Group x ROI	**77801**	**3.86**	**.02**	**< .001**	77801	0.53	.59	< .001	**77800**	**3.18**	**.04**	**< .001**
Emotion x ROI	77801	1.94	.14	< .001	77801	2.84	.05	< .001	77800	0.44	.64	< .001
Group x Emotion x ROI	77801	0.64	.53	< .001	77801	2.63	.07	< .001	**77800**	**3.32**	**.04**	**< .001**

Table 5. Grad-int task frequency domain analysis: Base and expression change response

	**Base response**				**Expression change response**			
	df	*F*	*p*	η_p^2	df	*F*	*p*	η_p^2
Emotion	1218	2.42	.12	< .001	1218	0.05	.82	< .001
Intensity	**1218**	**2.44**	**.04**	**.01**	**1218**	**2.86**	**.02**	**.01**
Group	43	1.94	.17	<.001	43	1.01	.32	< .001
ROI	**1218**	**8.34**	**> .001**	**.01**	1218	1.74	.17	< .001
Emotion x Intensity	1218	0.53	.71	< .001	**1218**	**3.44**	**.01**	**.01**
Emotion x Group	1218	1.40	.24	< .001	1218	0.58	.45	< .001
Intensity x Group	1218	0.62	.65	< .001	**1218**	**3.49**	**.01**	**.01**
Emotion x ROI	1218	0.31	.74	< .001	**1218**	**3.51**	**.03**	**.01**
Intensity x ROI	1218	0.34	.95	< .001	1218	1.42	.18	.01
Group x ROI	**1218**	**3.77**	**.02**	**.01**	1218	0.94	.39	< .001
Emotion x Intensity x Group	1218	1.24	.29	< .001	1218	0.60	.66	< .001
Emotion x Intensity x ROI	1218	0.37	.94	< .001	1218	0.08	.08	.01
Emotion x Group x ROI	1218	0.06	.94	< .001	1218	0.84	.84	< .001
Intensity x Group x ROI	1218	0.28	.97	< .001	1218	0.50	.50	.01
Emotion x Intensity x Group x ROI	1218	0.32	.96	< .001	1218	0.76	.75	< .001

Table 6. Grad-int task: Time domain analysis

	**Component 1**				**Component 2**				**Component 3**			
	df	*F*	*p*	η_p^2	df	*F*	*p*	η_p^2	df	*F*	*p*	η_p^2
Emotion	124877	3.05	.08	< .001	**124875**	**112.58**	**<.01**	**< .001**	**124869**	**16.56**	**<.01**	**< .001**
Intensity	**124868**	**7.72**	**< .01**	**< .001**	**124866**	**3.81**	**<.01**	**< .001**	**124862**	**42.26**	**<.01**	**< .001**
Group	42	0.20	.66	< .001	42	0.09	.77	< .001	42	0.03	.87	< .001
ROI	124846	0.06	.80	< .001	124846	1.24	.26	< .001	**124846**	**6.93**	**.01**	**< .001**
Emotion x Intensity	124873	1.05	.38	< .001	**124872**	**6.16**	**<.01**	**< .001**	**124867**	**3.66**	**.01**	**< .001**
Emotion x Group	124877	0.79	.37	< .001	**124875**	**11.90**	**<.01**	**< .001**	124869	0.92	.34	< .001
Intensity x Group	**124868**	**3.01**	**.02**	**< .001**	**124866**	**5.48**	**<.01**	**< .001**	124862	2.13	.07	< .001
Emotion x ROI	124846	0.19	.66	< .001	124846	0.01	.92	< .001	**124846**	**5.31**	**.02**	**< .001**
Intensity x ROI	124846	1.33	.25	< .001	124846	2.21	.06	< .001	124846	1.02	.39	< .001
Group x ROI	**124846**	**10.51**	**<.01**	**< .001**	124846	2.62	.11	< .001	**124846**	**11.57**	**<.01**	**< .001**
Emotion x Intensity x Group	**124873**	**4.33**	**.01**	**< .001**	124872	1.03	.39	< .001	**124867**	**5.56**	**<.01**	**< .001**
Emotion x Intensity x ROI	124846	0.71	.58	< .001	124846	1.22	.30	< .001	124846	1.32	.26	< .001
Emotion x Group x ROI	124846	0.50	.48	< .001	124846	2.06	.15	< .001	**124846**	**4.18**	**.04**	**< .001**
Intensity x Group x ROI	124846	1.41	.23	< .001	**124846**	**2.43**	**.04**	**< .001**	124846	1.94	.10	< .001
Emo. x Int. x Gr. x ROI	**124846**	**2.99**	**.02**	**< .001**	124846	1.31	.26	< .001	124846	1.22	.30	< .001

ADDITIONAL ANALYSIS S4: Grad-int task expression change with PDI correction

Table 7. Grad-int task frequency domain analysis: Grand-averaged Z-scores separated by emotion for each intensity threshold

**intensity**	**Z-score happy face at…**	**Z-score angry face at…**
20%	1.62	1.61
40%	1.61	1.44
60%	**2.05**	1.47
80%	**2.27**	**1.73**
100%	**2.16**	**2.24**

ADDITIONAL ANALYSIS S5: Grad-int task expression change with PDI correction

Table 8. Grad-int task frequency domain analysis: Expression change response corrected with a physical dissimilarity index (PDI)

	**Expression change response**			
	df	*F*	*p*	η_p^2
Emotion	1260	0.31	.58	< .001
Intensity	1260	2.11	.08	.01
Group	1260	0.32	.57	< .001
ROI	1260	0.07	.94	<.001
Emotion x Intensity	1260	1.78	.13	.01
Emotion x Group	1260	0.91	.34	< .001
Intensity x Group	1260	0.60	.66	<.001
Emotion x ROI	**1260**	**5.01**	**.01**	**.01**
Intensity x ROI	1260	0.69	.70	<.001
Group x ROI	1260	0.32	.72	< .001
Emotion x Intensity x Group	1260	0.11	.98	< .001
**Emotion x Intensity x ROI**	**1260**	**2.53**	**.01**	**.02**
Emotion x Group x ROI	1260	0.98	.38	< .001
Intensity x Group x ROI	1260	1.89	.06	.01
Emotion x Intensity x Group x ROI	1260	0.77	.63	< .001

References

Kirst, S., Diehm, R., Bögl, K., Wilde-Etzold, S., Bach, C., Noterdaeme, M., Poustka, L., Ziegler, M., & Dziobek, I. (2022). Fostering socio-emotional competencies in children on the autism spectrum using a parent-assisted serious game: A multicenter randomized controlled trial. *Behaviour Research and Therapy*, 104068. https://doi.org/10.1016/j.brat.2022.104068

Kirst, S., Zoerner, D., Schütze, J., Lucke, U., & Dziobek, I. (Eds.) (2015). *Zirkus Empathico: Eine mobile Applikation zum Training sozioemotionaler Kompetenzen bei Kindern im Autismus-Spektrum*. Pongratz, H. & Keil, R.
